# The Voluntary Hospitals and Legacy Duty

**Published:** 1913-01-25

**Authors:** 


### THE VOLUNTARY HOSPITALS AND LEGACY DUTY.

### Memorial to the Chancellor of the Exchequer.

The following memorial, dated January 15, 1913 (in which we have inserted cross-headings), signed by the Chairman and Honorary Secretary of the British Hospitals Association on behalf of upwards of 400 hospitals in the United Kingdom supported by voluntary contributions, has been prepared and forwarded to the Chancellor of the Exchequer by the British Hospitals Association.

Sir,—The Council of the British Hospitals Association, representing over 400 voluntary hospitals throughout the United Kingdom, begs your consideration to the following statement in reference to the work and financial position of these institutions, with a request that you will favourably entertain their claim to be relieved from the payment of duties to the Exchequer in respect of Legacy Duty upon bequests made to them.

### A Loss Not Only Direct.

The direct loss to the voluntary hospitals involved in the imposition of such a heavy burden as a 10 per cent. duty on their legacies is in itself a serious curtailment of the usefulness of the institutions; but the existence of such an impost operates indirectly to their prejudice in the minds of the benevolently inclined when making their testamentary arrangements. It is believed that if it were decided, through the wisdom of Parliament, that bequests to hospitals should hereafter be paid in full, the immediate result would be an increasing number of such benefactions to the benefit of the hospitals and the ultimate advantage of the State.

It is difficult to discern the principle on which the exemption granted to charitable institutions in Ireland from Legacy Duty, temporarily by the Stamp Duties (Ireland) Act, 1842 (5 and 6 Vic., cap. 82, sec. 38), and made permanent by the Stamp Act, 1853 (16 and 17 Vic., cap. 59, sec. 20), is withheld from similar institutions in England and Scotland. Your Memorialists feel that they may fairly and reasonably urge that hospitals and other charitable institutions in Great Britain should be placed on an equality in this respect with institutions of the same character and carrying out the same beneficent work in Ireland.

### A Relief Due to a National Work.

The Council submit that the assistance rendered to suffering patients by these hospitals is a national work, which, if not provided for by these institutions, would have to be undertaken by the State. The beneficence of the work, in not only relieving temporary suffering but in restoring patients to a state of health in which they can return to their several callings, and so resume the discharge of their duties to the State, to their employers, and to their families, has never been and cannot be called in question; while the importance of the public services they render as training schools for both the medical and nursing professions cannot be over-estimated.

Moreover, it is evident that the services of the hospitals will be needed as much after as before the passing of the National Insurance Act, inasmuch as the treatment afforded under the Act is only such as can be given by a general practitioner.

With all the voluntary hospitals the difficulty of meeting the progressive increase of expenditure is very great, and it is impossible to check altogether or even largely such continuing increase if the patients are to receive the benefits of the latest discoveries and improvements in medicine and surgery. These institutions are mainly dependent for their maintenance upon voluntary contributions and bequests; the total amount of their incomes derived from endowments being relatively very small as compared with their total expenditure.

### The Cheapest for the State.

Your Memorialists feel confidence in submitting that having regard to the voluntary duties discharged by hospitals supported by voluntary contributions, to the benefits received free from charge by large numbers of His Majesty's subjects whose sufferings and necessities demand and receive not only sympathy, but practical and most efficient relief, at great self-sacrifice on the part of many of the workers in their cause, it is incongruous that any portion of the benefactions to, or income of the hospitals, should be diverted from the objects for which they have been given, even as a contribution to the revenues of the State, bearing in mind that the moneys taken in taxation would otherwise be applied for purposes, the expense of which would fall on the State, if not voluntarily provided for by donors, subscribers, and workers.

Your Memorialists therefore respectfully but strenuously urge that hospitals supported by and mainly dependent on voluntary contributions should be exempted and relieved from payment to the revenue of duties on the benefactions made to them from time to time, the whole of which are expended in ministrations of compassion and mercy to those in suffering and extreme need.

### The View of the Profession.

From inquiries that we have made in London the view of the medical profession appears to be that the question will be decided by the Treasury on considerations of expediency. It is suggested by doctors that the Government is fully aware of the share of the financial burden of National Insurance which the hospitals are still taking voluntarily. If it be convinced therefore that the incidence of legacy duty will now prove sufficient to break down voluntary finance, it is argued that remission will be granted, for it is thought that the vast expense of a complete State hospital system is well recognised. On this view, it will be seen, the sum lost by remission of legacy duty on charitable bequests would be in the nature of a Government insurance against further expenditure.

